# Health communication is an epidemiological determinant: Public health implications for COVID-19 and future crises management

**DOI:** 10.34172/hpp.2022.28

**Published:** 2022-12-10

**Authors:** Alessandro Rovetta

**Affiliations:** ^1^R&C Research, Bovezzo (BS), Italy

## Dear Editor,

**Figure 1:**
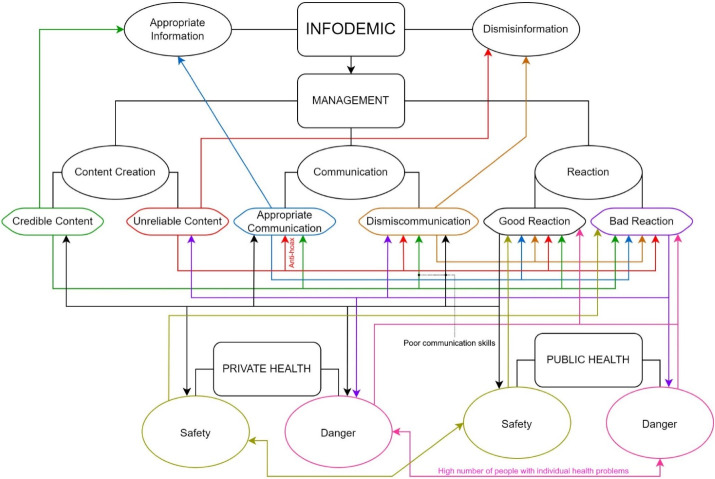
Summary infodemiological scheme of the main communication paths and information flow processing

 The World Health Organization (WHO) has defined COVID-19 as the most severe health emergency in history.^1^ Nonetheless, a primary epidemiological aspect is too often underestimated: a pandemic consists not only of a viral epidemic but also of an information epidemic.^2^ The latter is called “infodemic,” which is an overabundance of both correct and incorrect information.^3,4^ The damage of incorrect information (or dis-misinformation) was evident and striking during the COVID-19 crisis, ranging from the poisoning of US citizens due to the fake medical news uttered by President Donald Trump to the severe vaccination hesitancy due to the conspiracy hypotheses circulated on the web also thanks to some known scientists as the Nobel laureate Luc Montagnier.^2-5^ Despite much has been done by health authorities to counter it,^6^ the strategies adopted have often proved inadequate.^7^ In this regard, it should be emphasized that risk communication and community engagement are subject to significant challenges such as distrust in government, cultural, social, and religious resistance, and inertia.^8^ Besides, the variety of pathways, channels, and methods of communication – including mass and social media, online sharing platforms, and messaging apps – have made the flow of health information uncontrollable.^9^ Nevertheless, this editorial primary focuses on a more subtle aspect of the matter, i.e., the involuntary sharing of unreliable news (we call it “type 1 misinformation” or “T1M”) and the sharing of truthful news with a negative communicative outcome (we call it “type 2 misinformation” or “T2M”). T1M is mainly due to human errors, which include interpretations, calculations, data collection, or language abuse. Although circulating information will inevitably increase rapidly during times of health crisis, some scientists suggest this phenomenon can be reduced by limiting indiscriminate access to non-peer-reviewed material or classifying the content’s reliability through a color scale easily understandable by the lay public.^3,10^ On the contrary, T2M is due to a more complex combination of factors, such as information and risk perception and communication skills. Indeed, how an individual processes incoming information depends on deep unconscious dynamics determining his/her reaction and response behavior.^11,12^ Scientists and researchers are used to sharing their opinions or findings via mass media like social networks and television, often without having the communication expertise to do it properly, i.e., to consider the above psychological determinants and structure their message based on them. While this may be relatively safe regarding issues with little immediate impact (e.g., the discovery of a new black hole), such misconduct can become extremely dangerous if delicate public health issues are addressed (e.g., vaccines, therapies, and drugs). In particular, this happened more frequently during COVID-19.^3^ In addition, the media’s obsession with breaking news led to a lack of coherence and poor self-efficacy in news coverage of COVID-19.^13^ Therefore, it is fair to conclude that public figures (including celebrities, news presenters, and politicians), journalists, and even scientists have contributed directly to such a dramatic infodemiological scenario. However, this consideration must not be made to blame but to become aware that communication is one of the most significant epidemiological factors.^7^ Furthermore, the scientific community must take into account that an infodemic is also composed of correct information since the overload of information can lead to confusion, fear, and negative responses as well.^14^ Ergo, reacting to dis/misinformation with correct information is not a sustainable long-term strategy. Figure 1 shows the enormous complexity of the infodemiological process. The key points are as follows:

- Information with reliable content can feed the infodemic if communicated by non-communication professionals, even if they are respected scientists or researchers.
- Even well-presented information with valid content can foment infodemic due to information overload, functional illiteracy, and unconscious mechanisms that are difficult to model and predict.^3,8,11-16^
- The only relevant aspect of infodemiological science is also the central aspect of epidemiological science, that is, people’s behavior and reaction.
- Since all the variables influence each other (complex system), it is difficult to predict a long-term outcome.

 According to the above points and the WHO suggestions,^2,3^ it is clear that no information campaign alone can counteract the infodemic phenomenon. Consequently, we must insist on creating resilience to dis/misinformation and information management abilities in the lay public. Moreover, it is equally clear that expert communicators must perform communication and that scientists should only mediate the content to be communicated. Indeed, especially during an epidemic, it is vital to stress that public response is the major epidemiological determinant.^3,7,17^ For example, non-compliance with containment regulations and vaccination hesitancy have been a health scourge because of their direct damage (e.g., unvaccinated exposed people) and indirect effects (e.g., overcrowding of health facilities).^18^ Hence, the infodemiological approach regarding communication is primary and cannot be ignored or underestimated. Specifically, any public health management strategy during a health crisis must put communication – intended both as the disclosure and receipt of health information – at the same level as direct interventions since the public response is equally critical to its success. Besides, the authors of this paper emphasize the importance of establishing a school educational program that introduces children to these issues, accustoming them to the choice and emotional management of the flow of information.^3^ In fact, such preparation takes time to be assimilated – which requires action as soon as possible to avoid future infodemics – and is essential to ensure a good response from the public. In this regard, it is necessary to consider that the psychological damage of an infodemic does not translate into epidemiological damage only through the inadequate concrete actions of the population (indirect damage) but also through the immune and cardiovascular systems’ negative response (direct damage). For instance, anxiety has emerged as one of the most important risk factors for cardiovascular disease,^19^ while chronic stress can markedly lower immune defenses.^20^ These health consequences may further burden health systems already compromised by the epidemic emergency, generating a vicious circle of cause and effect due to the lack of medical assistance. In conclusion, communication, understood as a combination of dissemination and reception of information, is one of the main epidemiological factors since it determines the population’s behavior. The infodemiological scenario that characterizes a health crisis is complex and involves non-intuitive psychological mechanisms. Especially during the COVID-19 pandemic, this aspect was neglected, and information was often poorly shared by people not competent in the disclosure matter. As a consequence, too many people have disrespected and protested against basic public health precautions.^7^ The lesson to be learned is that there can be no epidemiology without infodemiology. The propagation of information is as impactful as that of an infectious agent since the public response drives the evolution of the related infection and health crises. The absence of an infodemiological plan, together with epidemiological errors, has been the cause of suffering and millions of avoidable deaths. From now on, without further delay, governmental authorities worldwide must invest resources in the infodemiological training of scientists and the lay public. For these reasons, the authors of this paper suggest including communication experts in managing health crises, setting up mandatory communication courses for all scientists with a disclosure role, and immediately starting a school education program to create infodemic resilience, critical scientific sense, and emotional management skills for incoming information excess. In this regard, non-communications scientists are called to a humble admission of ignorance in the name of ethics that must guide scientific action.

## Acknowledgements

 I thank Lucia Castaldo for her support and the infodemiological contribution.

## Funding

 This research did not receive funding.

## Ethical Approval

 Not applicable.

## Competing Interests

 The author declares that he has no conflicts of interest.
